# Neuroscience and human nature: Review of *The Altruistic Brain*

**DOI:** 10.3389/fpsyg.2015.00307

**Published:** 2015-03-18

**Authors:** Patrick C. Trettenbrein

**Affiliations:** ^1^Language Development and Cognitive Science Unit, Department of English Studies, University of GrazGraz, Austria; ^2^Department of Linguistics, University of GrazGraz, Austria

**Keywords:** morality, altruism, Altruistic Brain Theory, linguistic analogy, moral psychology

As a rule, a natural scientist writing on an evidently philosophical subject matter sets the average philosopher's antennae quivering. Nevertheless, (cognitive) neuroscience has matured as a field of research and its practitioners are now ready and able to interpret the consequences of their research for our understanding of the individual as well as society as a whole. In *The Altruistic Brain* Donald Pfaff sets out to and succeeds in doing just this when he puts forward what he calls Altruistic Brain Theory (ABT). Essentially, he postulates that findings from neuroscience indicate that as human beings we are “wired” to be good, just as we are “wired” to acquire natural language(s). Pfaff's book, which is written for a non-specialist audience, thus presents and interprets evidence in favor of an idea already expressed by Wilhelm von Humboldt who declared that humankind is intrinsically more inclined to philanthropic than self-serving actions (von Humboldt, 1792/1851, p. 98).

The book is divided into two parts, the first one introducing ABT, the second part being a discussion of the theory's implications for individual behavior, society as a whole, and jurisprudence. Intriguingly, ABT is simple insofar as that it posits that the brain processes altruism in only five steps, all of which are rooted in well-understood neurocognitive mechanisms. Pfaff cites neurophysiological studies showing that the brain represents actions before they are taken (step 1), as well as neuroimaging studies indicating, for example, a dedicated “face recognition module” for representing the target of a potential action (step 2). Vision is used as an example throughout the book whereas the author makes it clear that other modalities can be expected to be involved as well. For step 3, the author relies on the cross-excitation of neurons to argue that representations of the “target” and the “self” are being merged before the emotional consequences of the impending action are evaluated and presented to a supposed “ethical switch” in the circuitry between the amygdala and the prefrontal cortex (step 4). Finally, step 5 then constitutes a straightforward yes-no decision which Pfaff locates in the insula. Whether an altruistic action is ultimately carried out furthermore depends on neural and hormonal mechanisms that, as Pfaff holds, have evolved to promote prosocial behavior.

ABT and its implications diverge from the commonplace argument for human self-interest that is so prevalent in many parts of the (social) sciences. Hence, the book proceeds by asking and attempting to answer the most pressing question resulting from ABT: “If there is such as mechanism, then we are all *equipped* to be good, and the overarching question becomes: how do we create conditions that favor our natural inclinations, and limit impulses that undermine such inclinations?” (p. 141, emphasis in original). Consecutively, Pfaff discusses the implications of ABT in regard to how we might think of ourselves and others, as well as what the theory means for how we can understand and address bad behavior of individuals. In regard to the societal level, the author outlines how individual bad behavior might multiply in a social setting (e.g., the negative group dynamics of gangs) and what could be done to prevent this, only to ultimately conclude that, it comes as no real surprise, there are no easy answers. While ABT expresses a universal tendency it is, unfortunately, not an absolute.

Throughout, Pfaff's writing is very accessible to the non-specialist, whenever he employs technical terms and concepts from neuroscience, genetics, biology, or anthropology he makes sure to at least briefly introduce them to the reader. Much more important than the style in which it is written, the book provides one of the first—if not the very first—compilation of evidence from primary neuroscience research in favor of such a universal altruistic predisposition as a central aspect of human nature as described by ABT. Hence, the book's clear strength and simultaneously its key feature is to be found in the first part, when the author presents his argument by discussing and reinterpreting neuroscientific findings in favor of his theory that would otherwise have appeared to be solid yet generic research. In combination with the accessibility to the general reader, Pfaff has done a magnificent job in compiling, reinterpreting, and presenting the neuroscientific evidence available as of today.

Because he is writing for a non-specialist audience and the public at large, Pfaff's style of writing—despite being refreshingly casual—is more often repetitive and appears “over-structured” when he continuously points the reader to what he has said in the previous chapter, is going to say now, and will consider only later in the book. Putting stylistic features aside, another issue that arises from reading the book is the author's insistence on his idea of ABT as something very novel. Even though he refers to the prominent linguist and founding father of modern linguistics, Noam Chomsky, several times in the book, Pfaff fails to make a connection with work in the cognitive sciences that has followed this train of thought by drawing an analogy with modern linguistics (e.g., Mikhail, 2007; Dwyer et al., 2010). How Pfaff's work fits into the greater picture of this research program certainly would have been of interest to the gentle reader. All in all, the general idea that the human brain is “wired” for altruism just as it “wired” for language acquisition is thus arguably not so new.

To summarize, *The Altruistic Brain* presents and discusses evidence from neuroscience with far-reaching consequences and implications for how we conceive of human nature and is therefore of interest to anyone interested in the study of morality in the widest sense. Due to the breadth and range of consequences that the idea of humans having a predisposition for altruistic behavior implicates, the book should be obligatory reading not just for (cognitive) scientists and (moral) philosophers interested in the subject matter but everyone who is involved in decision-making processes in regard to social and legal policy. Donald Pfaff's book provides its readers with scientific evidence for Wilhelm von Humboldt's intuitive assessment and thereby highlights that knowledge about human nature stemming from the natural sciences can and should inform how we think about our species and the organization of our societies. When the Bible has depicted us as original sinners for so long, it seems to be about time for a change of perspective.

## Conflict of interest statement

The author declares that the research was conducted in the absence of any commercial or financial relationships that could be construed as a potential conflict of interest.
